# SHORT-TERM U.S. PATHS TOWARD RETIREMENT: SIMILARITIES AND DIFFERENCES ACROSS AGE-GENDER-CLASS INTERSECTIONS

**DOI:** 10.1093/geroni/igz038.2963

**Published:** 2019-11-08

**Authors:** Sarah Flood, Phyllis Moen, sarah flood, Janet Wang

**Affiliations:** University of Minnesota, Minneapolis, Minnesota, United States

## Abstract

Existing knowledge about retirement transitions comes from studies of cohorts living through demographic, technological, social and economic environments, and private sector and public policy regimes that are very different from those of today. The Boomer cohort now transitioning to retirement is more educated, healthier, and more engaged in paid work than their parents or grandparents at the same ages. How is the large, diverse Boomer cohort (in their 50s, 60s, and 70s) navigating leaving the labor force compared to the Silent Generation preceding them? How similar or different across cohorts are patterned yet heterogeneous short-term workforce pathways, in terms of the timing, sequencing, and voluntariness of working hours, work participation, and exits, including subjective retirement? We use linked Current Population Survey (CPS) panel data (over 16 months) to capture change, complexity, heterogeneity, and inequities in even short-term work/retirement dynamics across intersections of age, gender, and class.

